# Supercritical CO_2_ Assisted TiO_2_ Preparation to Improve the UV Resistance Properties of Cotton Fiber

**DOI:** 10.3390/polym14245513

**Published:** 2022-12-16

**Authors:** Sihong Ye, Hui Sun, Juan Wu, Lingzhong Wan, Ying Ni, Rui Wang, Zhouyang Xiang, Xiaonan Deng

**Affiliations:** 1 Cotton Research Institute of Anhui Academy of Agricultural Sciences, Hefei 230009, China; 2 State Key Laboratory of Pulp and Paper Engineering, South China University of Technology, Guangzhou 510640, China

**Keywords:** cotton fiber, TiO_2_, supercritical carbon dioxide, UV resistance

## Abstract

Cotton fiber is favored by people because of its good moisture absorption, heat preservation, soft feel, comfortable wearing and other excellent performance. In recent years, due to the destruction of the ozone layer, the intensity of ultraviolet radiation at ground level has increased. Cotton fiber will degrade under long time ultraviolet irradiation, which limits the outdoor application of cotton fiber. In this study, titanium dioxide (TiO_2_) particles were prepared on the surface of cotton fibers with the help of supercritical carbon dioxide (SCCO_2_) to improve the UV resistance of cotton fibers. The effects of SCCO_2_ treatment on the morphology, surface composition, thermal stability, photostability and mechanical properties of TiO_2_ were studied by Fourier transform infrared spectroscopy, X-ray diffraction, X-ray photoelectron spectroscopy, scanning electron microscopy, thermogravimetric analysis, UV-VIS spectroscopy, and single fiber test. The results showed that TiO_2_ particles were generated on the fiber surface, which reduced the photo-degradation rate of cotton fiber. This is because TiO_2_ can absorb UV rays and reduce the absorption of UV rays by the cotton fiber itself. The synthesis process of SCCO_2_ is simple and environmentally friendly, which provides a promising technology for the synthesis of metal nitrogen dioxide on natural plant fibers.

## 1. Introduction

Cotton fabric has excellent moisture absorption, heat preservation and air permeability, which is widely favored by people, and is also widely used in medical treatment and various high-end clothing products. In recent years, because the ozone layer is destroyed gradually, the ultraviolet radiation on the ground increases. Under ultraviolet irradiation, cotton fiber is prone to photo-oxidation reaction and aging phenomenon, which not only affects the color of the fabric, but also seriously affects the mechanical properties of the fabric, reduces the wear resistance of the fabric, and increases the ultraviolet radiation of human skin [1].The US Environmental Protection Agency estimates that ozone depletion will cause some new negative health effects by 2075, such as accelerated skin aging, photo dermatosis, erythema, and severe skin cancer [2]. Therefore, the need to provide UV protection is strong, and cotton textiles play an important role in this regard.

The anti-photoaging property of cotton fiber can be improved by adding antioxidants on the surface of cotton fiber. Titanium dioxide (TiO_2_), which has high chemical stability, high refractive index [3,4,5,6], non-toxicity and strong light catalytic activity, has attracted much attention in recent years due to its potential applications in the energy and environmental fields [7,8]. TiO_2_ has excellent UV shielding, infrared absorption and photocatalytic properties, and its application on cotton fibers can give cotton fibers a variety of functions such as antibacterial, self-cleaning, and UV protection. As a general liquid-phase based method for the synthesis of particles and inorganic network oligomers, sol-gel method has great advantages in the hydrolysis and condensation of the precursor to obtain nanoTiO_2_ [9], and has been widely used in hybrid coating materials [10]. Due to the easy preparation of sol-gel method has attracted extensive attention in the synthesis and modification of materials. It is worth mentioning that the TiO_2_ prepared by sol-gel method can modify the cotton fiber and improve the anti-photoaging performance of the cotton fiber [11]. Unfortunately, when the sol-gel method is applied to the fiber surface modification, it is found that the adhesion is low, easy to fall off, and affect the mechanical properties of cotton fiber. Liu [12] used a two-step method to grow SiO_2_/TiO_2_ multilayer films on the surface of cotton fibers by sol-gel method. The results showed that growing dense and uniform multilayer on fiber surface could improve the outdoor application performance of cotton fiber, but the adhesion of the surface of the fiber was poor. Bai [13] designed a natural acid-alkali resistant TiO_2_ coated cotton fabric by single-side immersion method, while the TiO_2_ coating prepared by the sol-gel method is brittle and has poor durability.

Supercritical carbon dioxide (SCCO_2_) has long been considered as the green medium of polymer chemistry, which has stable chemical properties [14], non-toxic, non-corrosive, and carrying effect [15,16,17,18,19]. At the same time, the critical temperature of supercritical carbon dioxide is 31.1 °C and the critical pressure is 7.37 MPa, so the conditions of SCCO_2_ are easy to achieve [20]. The density of SCCO_2_ is close to that of liquid, so it has comparable solubility and solute carrying capacity with liquid [21,22], and plays an increasingly important role in the surface modification of polymer [23,24,25,26]. Fortunately, JoabelRaabe deposited particles on cellulose fibers by sol-gel method and obtained organic-inorganic hybrid materials by chemical bonding [27]. L.Z. reported a method to synthesize particles on the surface of fiber to improve its UV resistance in SCCO_2_ [28]. Particles are not easy to fall off and the mechanical properties of fibers are greatly improved. Through the swelling and carrying properties of SCCO_2_, Kong [29] carried HDI to the surface of the fiber and grafter into it. The results show that the strength and modulus of the modified fiber are improved. Therefore, surface grafting with SCCO_2_ can not only reduce the damage of the fiber caused by solvent and enhance the roughness of the polymer fiber surface, but also improve the surface properties and increase the mechanical properties of the fiber.

In this paper, CF-TBT modified cotton fiber was prepared by loading titanate (TBT) precursor on the surface of cotton fiber with SCCO_2_ fluid. Then, the protoplasts were further hydrolyzed in situ to become TiO_2_ photodegradation agent, and CF-TiO_2_ modified cotton fibers were obtained. The effect of SCCO_2_ assisted TiO_2_ preparation on the surface of cotton fiber on its surface morphology, crystallinity, and structure was studied. The effect of SCCO_2_ modification on the mechanical properties and UV resistance of cotton fiber was also studied.

## 2. Materials and Methods

### 2.1. Materials

Cotton fibers were supplied by Anhui Jingxiu Textile Company Co., Ltd. (Xuancheng, China). Carbon dioxide (purity: +99.99%) was purchased from Shandong Yong Cylinder Co., Ltd. (Linyi, China). Tetra butyl titanate (TBT, purity: 99%) was purchased from Macklin Biochemical Co., Ltd. (Shanghai, China). Acetone (purity: +99.5%) was bought from Shanghai Yunli Trading Co., Ltd. (Shanghai, China). Anhydrous ethanol (purity: +99.7%) was bought from Sinopharm Chemical Reagent Co., Ltd. (Changshu, China). Acetic acid (purity: +99.7%) was bought from Jiangsu Qiangsheng Functional Chemical Co., Ltd. (Changshu, China). These chemicals were used without further purification.

### 2.2. Cotton Fiber Degreasing

Degreasing experiments were carried out on cleaned cotton fibers by adding 400 mL NaOH and 0.08 g osmolyte to 80 g cotton. The degreasing work is divided into three stages: the first stage is heating up, heating up to 90 °C for 30 min; The second stage was the heat preservation stage, and the temperature was controlled at 90 °C for 50 min. In the third stage, warming was continued at 100 degrees for 10 min. The fourth stage is cooling down.

### 2.3. Synthesis of TiO_2_ in SCCO_2_

**Figure 1:**
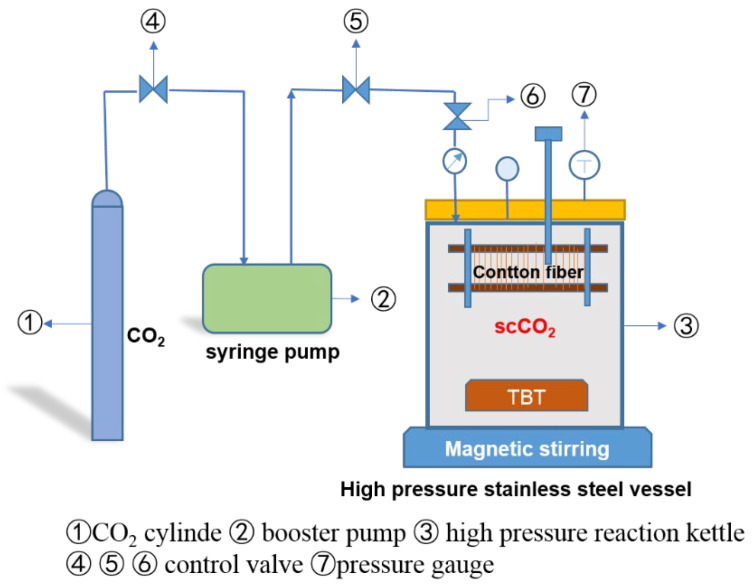
The scheme of theSCCO_2_ treatment device.

The experiment was carried out in a stainless-steel supercritical reactor, which was equipped with a booster pump to push CO_2_ into the reactor. The reactor device is shown in Figure 1.

**Figure 2:**
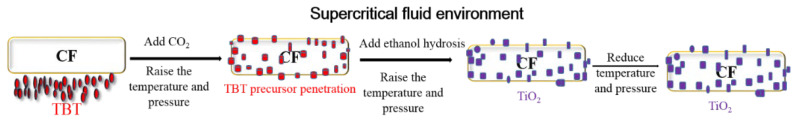
The process of synthesizing TiO_2_ for CF in SCCO_2_.

TiO_2_ preparation on cotton fiber surface is divided into two steps:

(1) 20 mL of TBT precursor was placed on the bottom of the reactor, and then the defatted cotton fiber was placed on the stainless steel frame in the reactor to separate it from the TBT precursor. The temperature was adjusted to 100 °C and pressure was set to 10 MPa, and the response time was set for 2 h. Because supercritical CO_2_ fluid (SCCO_2_) has the function of carrying, swelling and dissolving, TBT can be dissolved and carried to the fiber surface, and the resulting fiber is called CF-TBT.
(2) 30 mL ethanol, 0.1 mL glacial acetic acid, and 3 mL deionized water were mixed and stirred at the bottom of the reactor, and the CF-TBT fiber obtained in the first step was placed on the stainless steel frame in the reactor. Adjust the temperature to 100°C and pressure to 10 MPa again, and the reaction time was 2 h. TiO_2_ was prepared on the fiber surface by in situ hydrolysis of the precursor TBT deposited on the fiber surface. After the reaction, the fibers were washed with acetone, ethanol, and deionized water and dried, and the resulting fiber is called CF-TiO_2_.The schematic illustration of a two-step process is shown in Figure 2.

### 2.4. Characterizations

**Figure 3:**
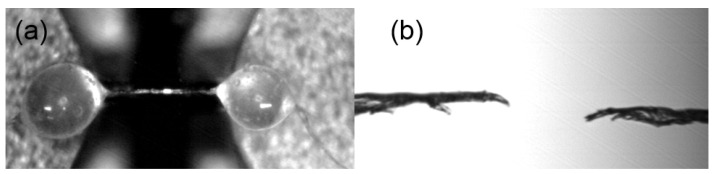
Fiber mechanical properties measurement chart: (**a**) pretension state; (**b**) fracture state of fiber.

The surface functional groups were studied using Fourier transform infrared spectroscopy (FTIR) with a diamond attenuated total reflectance. The surface morphology and chemical compositions of the CF and CF-TiO_2_ were determined. The morphology of the pretreated cotton fibers was observed using a JEM-6390LV scanning electron microscope (SEM) (JEOL, Tokyo, Japan) at 15 kV. The samples were coated with platinum using a vacuum sputter coater before they were observed. X-ray diffraction (XRD, Smartlab9Kw, Tokyo, Japan) was used to determine the crystalline phase of CF and CF-TiO_2_. The measurement was conducted with a Cu Kα radiation source (λ = 1.5406 Å) with 40 kV and 200 mA), followed by a scanning range of 5.0°–90.0° at a speed of 5°/min. A UV–visible (UV-Vis) spectrophotometer (UV3600, Shimadzu, Japan) equipped with an integrating sphere was used to measure the absorbance of the fibers in the wavelength range of 200–450 nm. The cotton fibers were cut into powders. Then, the powder of the fiber was pressed into a thin layer at the center of the sphere and the measurements were performed. A thermogravimetric analyzer (TGA550, TA, New Castle, DE, USA) was used to measure the thermal stability of the fibers. Temperature ramp measurements were conducted under a nitrogen atmosphere from 30 to 900 °C at 20 °C·min^−1^. A simple artificial accelerated aging tester was built with a UV lamp (UVB 280–315 nm, 40 W, lamp length of 1220 mm, Baoxiang Lighting Technology Co. Ltd., Guangzhou, China) to study the effect of UV aging over time on the mechanical properties of the fibers. The distance between the fiber sample and the UV lamp was 20 cm. The fibers were placed in parallel rows in the sample tray which was exposed to 40 W/m^2^ and held at a relative humidity of 60% for 192 h, according to the Chinese Standard GB/T 14522-93. The mechanical properties of single fibers of cotton fiber were measured by high-precision plant staple fiber mechanical properties tester (JSF08, Powereach, Shanghai, China). The tensile strength and elastic modulus of single fiber were measured. The measuring range of the sensor is 980.7 mN, the tensile load accuracy is 0.01 μN, and the tensile rate is 0.05 mm /min. The tensile strength, elastic modulus, and elongation at break were calculated according to the formula in GB/T 35378-2017. The results were analyzed by Excel and origin software. Figure 3 is the measurement chart of fiber mechanical properties, Figure 3a is the top view under 3.5 magnification under pretension, and Figure 3b is the front view under 1 magnification.

## 3. Results and Discussion

### 3.1. Influence of the Treatment on the Structures of Fibers

#### 3.1.1. FTIR Analysis

**Figure 4:**
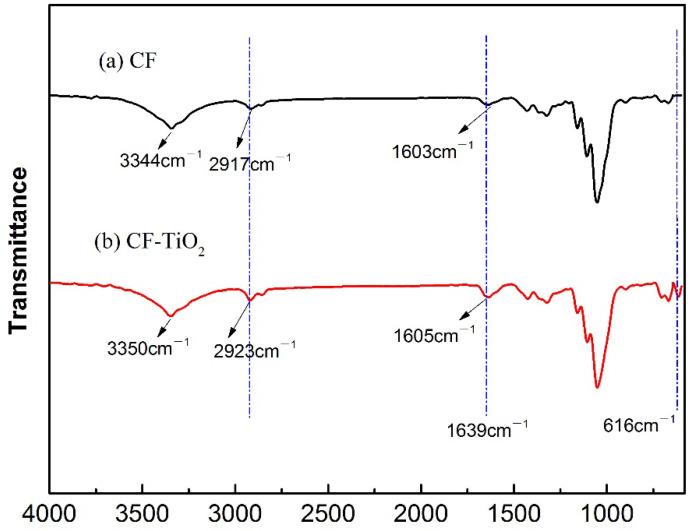
FTIR curves: (**a**) CF; (**b**) CF−TiO_2_.

In order to study the binding mode of TiO_2_ particles and cotton fiber, Fourier infrared spectroscopy ATR method was used to further characterize CF and CF-TiO_2_ samples. Curve a in Figure 4 is the infrared spectrum of raw cotton fiber, and curve b is the infrared spectrum of CF-TiO_2_.The peak value at 3344 cm^−1^ is the intramolecular hydrogen bond stretching vibration of cellulose in CF. The peak value at 2917 cm^−1^ is the asymmetric stretching vibration of CH_2_ of non-cellulose of CF [30]. The H-O bending vibration peak is at 1639 cm^−1^.

The vibration peak at the corresponding position of curve a in Figure 4 can also be observed at the same position of curve b, and the vibration peak intensity of 1063 cm^−1^, 2917 cm^−1^ and 3344 cm^−1^ is obviously enhanced and tends to shift towards the higher wave number. This is because in the process of TiO_2_ synthesis on the surface of cotton fiber, a large number of hydroxyl groups contained on the surface of TiO_2_ formed hydrogen bonds with the hydroxyl groups on the surface of CF, which strengthened the intensity of the vibration absorption peak of CF-TiO_2_ [31]. At the same time, the supercritical-treated cotton fiber (Figure 4b) showed a new absorption peak at 616 cm^−1^ [32] compared with the untreated CF (Figure 4a), which was just within the Ti-O bond absorption peak interval, indicating that TiO_2_ particles were successfully prepared on the surface of the cotton fiber.

#### 3.1.2. SEM Analysis

**Figure 5:**
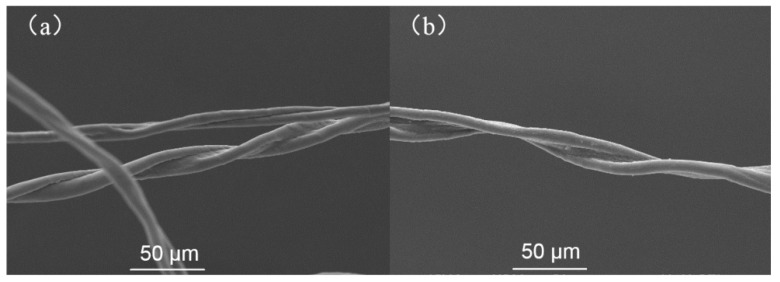
SEM images: (**a**) CF; (**b**) CF-TiO_2_.

The surface morphology of cotton fiber was studied by scanning electron microscope. SEM images of CF and CF-TiO_2_ are shown in Figure 5. It can be seen that the untreated fibers have a smooth and clean surface. Compared with untreated CF, the surface of modified CF-TiO_2_ (Figure 5b) is rough with many obvious particles on the surface.

**Figure 6:**
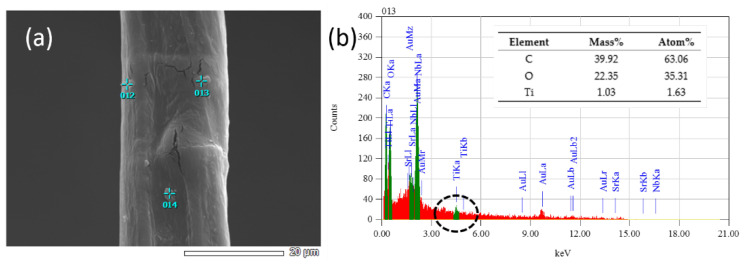
(**a**) SEM images ofCF-TiO_2_; (**b**) EDX-mapping images of CF-TiO_2_.

In order to further determine the distribution of TiO_2_ on the surface of cotton fabric, SEM combined with EDX-mapping was used to analyze the surface of CF-TiO_2_ (Figure 6). It can be obviously observed from Figure 6a that there are particles on the surface of cotton fiber. We performed anEDX analysis of particle number 13 in Figure 6a and confirmed that the particle was TiO_2_ from the energy chromatogram. The Ti peak in Figure 6b was not obvious due to the relatively low TiO_2_ load. XPS analysis further confirmed the existence of Ti atoms on the surface of modified cotton fiber.Combined with the FTIR analysis, we can conclude that these particles are TiO_2_.

#### 3.1.3. Crystalline Structure

**Figure 7:**
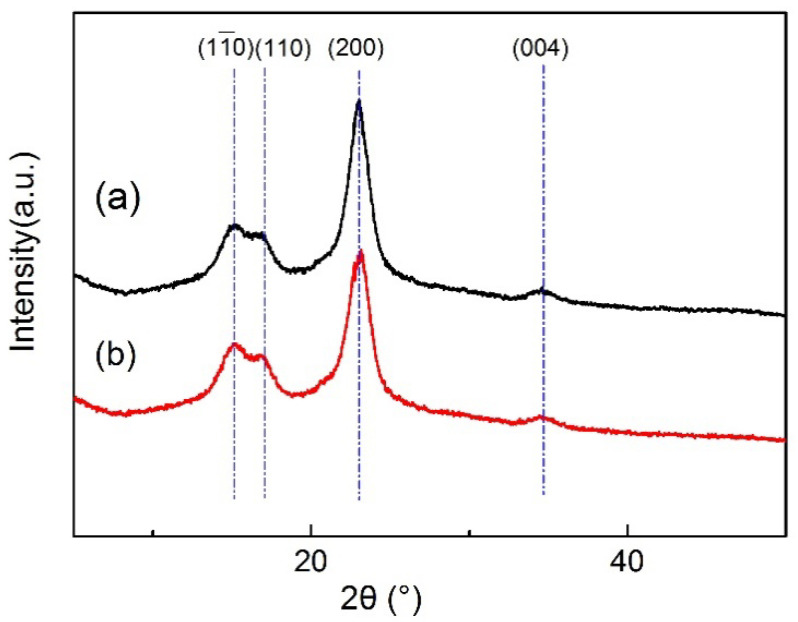
XRD patterns: (**a**) CF; (**b**) CF-TiO_2_.

Figure 7 shows the XRD patterns of CF and CF-TiO_2_ fibers. It can be clearly seen from Figure 7 that cotton fiber has four diffraction peaks between 10° and 50°. These four diffraction peaks represent four crystal planes, which are (110), (110), (200), and (004), and the diffraction angles are 14.82°, 16.38°, 22.68°, and 34.61°, respectively, which constitute the typical XRD pattern of cotton fiber [33]. It can be seen that (200) crystal plane has the strongest diffraction intensity and (004) crystal plane has a weak intensity. XRD curve on CF-TiO_2_ (Figure 7b) samples found no crystalline phase of TiO_2_. At the same time, compared with untreated CF, the crystallinity of cotton fiber of CF-TiO_2_ sample decreased, indicating that the TiO_2_ formed on the fiber was amorphous.

#### 3.1.4. XPS Analysis

**Figure 8:**
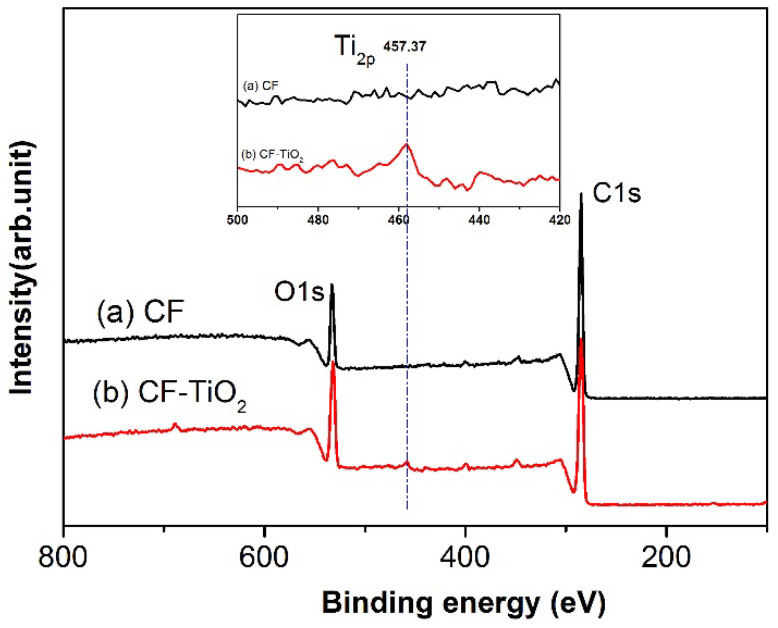
XPS spectrum of untreated CF (**a**) and CF-TiO_2_ (**b**).

In order to study the effect of supercritical fluid treatment on the surface of cotton fiber, the surface components of CF and CF-TiO_2_ were determined by XPS method. The results showed that the surface oxygen content of CF-TiO_2_ fiber was higher than that of untreated CF. As shown in Figure 8, C1s and O1s peaks appear at 284.8 and 531.6 eV, respectively. The CF-TiO_2_ fiber shows a new peak of titanium atoms at 457.37 eV compared to the untreated CF. The result of element content change is shown in Table 1. Compared with untreated CF, the content of C in CF-TiO_2_ decreased, the content of O and Ti increased, and the ratio of O: C increased from 0.1780 to 0.2705. The content of Ti reached 1.2%, indicating that TiO_2_ was successfully prepared on the surface.

#### 3.1.5. Mechanical Properties

Mechanical properties of CF and CF-TiO_2_ samples are shown in Table 2. The tensile strength and modulus of CF-TiO_2_ treated with SCCO_2_ increased by 6.37% and 7.80% compared with the untreated CF samples. The reason for this phenomenon may be that SCCO_2_ fluid has a relatively strong washing effect, which can remove some impurities on the surface of cotton fiber. At the same time, SCCO_2_ fluid can enhance the movement of the chain segment of cotton fiber, and further optimize the structure of the molecular chain of cotton fiber under the action of certain pressure and temperature. To a certain extent, the structural defects of the fiber are reduced, and the mechanical properties of cotton fiber are improved.

### 3.2. UV-Resistance Analysis

#### 3.2.1. SEM Analysis

**Figure 9:**
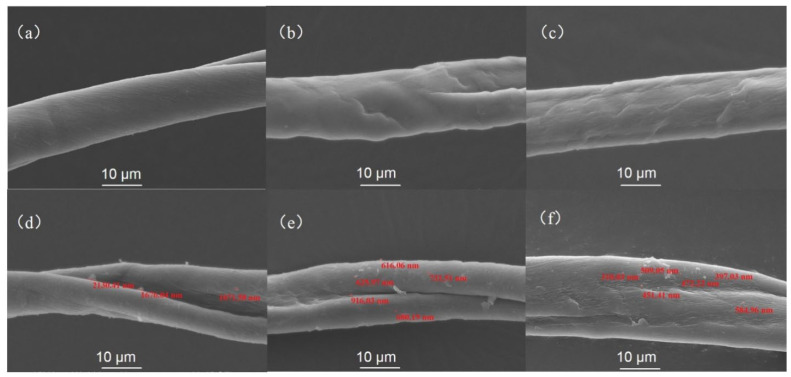
SEM images of samples after UV aging: (**a**) CF; (**b**) CF-UV-48 h; (**c**) CF-UV-196 h; (**d**) CF-TiO_2_; (**e**) CF-TiO_2_-UV-48 h; (**f**) CF-TiO_2_-UV-196 h.

Figure 9 shows the microscopic morphology (SEM) of unmodified CF and CF-TiO_2_ after 48 h and 196 h photo-aging. It can be seen that the surface of untreated CF (Figure 9a) is relatively smooth. After accelerated aging with UV light for 48 h, the surface of untreated CF (Figure 9b) becomes rough with a small amount of etching and streaking. After 196 h accelerated aging, the untreated CF (Figure 9c) surface became rougher, with more etchings, furrows, and streaks. However, after 48 h accelerated aging of the treated CF-TiO_2_ (Figure 9d) sample, the surface of sample (Figure 9e) did not show obvious changes, and after 196 h accelerated aging of sample (Figure 9f), there were only some fine lines on the surface, without the above obvious etching and groove. Meanwhile, as shown in Figure 9, TiO_2_ particles gradually become smaller with the continuous absorption of UV. Therefore, SCCO_2_ assisted in situ preparation of TiO_2_ on the surface played a role in slowing down photoaging of cotton fibers and played a protective role on fibers.

#### 3.2.2. UV-Vis Analysis

**Figure 10:**
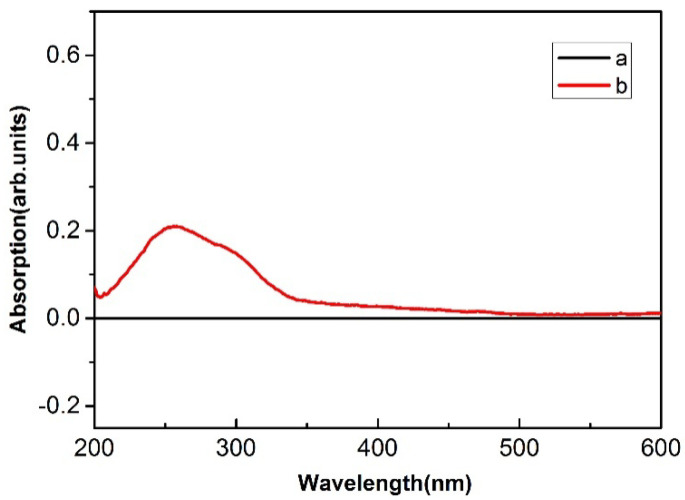
UV−Vis curves: (**a**) CF; (**b**) CF−TiO_2_.

As can be seen from Figure 10, the UV-Vis spectrum of CF is in the range of 200–600 nm, in which 250–400 nm is the ultraviolet absorption region and 400–600 nm is the visible absorption region. Compared with untreated CF, the UV absorption effect of treated CF-TiO_2_ was significantly improved. CF-TiO_2_ samples have strong absorption at 200–400 nm, but almost no absorption at 400–600 nm. As can be seen from the figure, the absorption value of CF-TiO_2_ sample at 258 nm was increased by 21.51% compared with untreated CF. The reason for this phenomenon is as follows: the electronic structure of TiO_2_ is characterized by an empty conduction band and a full valence band. The gap between the conduction band and the valence band is 3.0 eV, which is equivalent to the energy of a photon with a wavelength of 413 nm. When the wavelength of TiO_2_ is not higher than 413 nm, the electrons in the valence band will absorb energy and produce a transition, forming two kinds of carrier fluids, thus playing the role of UV shielding [34].

#### 3.2.3. Mechanical Properties

**Figure 11:**
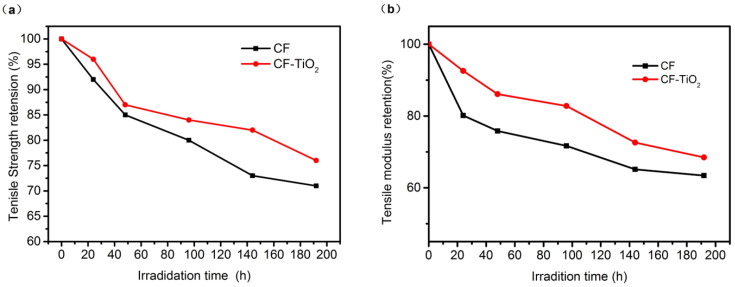
Mechanical properties of CF and CF-TiO_2_ after UV irradiation: (**a**) Tensile Strength retention (%); (**b**) Tensile modulus retention (%).

The CF and CF-TiO_2_ samples were subjected to UV aging for different times, and the mechanical properties of the fibers before and after modification were studied. Figure 11 shows the strength and modulus mechanical properties of CF and CF-TiO_2_ samples after UV aging for 24 h, 48 h, 96 h, 144 h, and 196 h. With the increase of photoaging time, the strength and modulus of cotton fiber showed a downward trend, which indicated that ultraviolet radiation caused damage to the mechanical properties of cotton fiber. However, the decreased degree of CF-TiO_2_ samples is lower than that of untreated CF, indicating that the preparation of TiO_2_ on the surface of cotton fiber can effectively slow down the fiber photoaging rate.

It can be seen from Figure 11 that the tensile strength and modulus retention rates of CF-TiO_2_ samples after UV irradiation for 196 h are 76.53% and 68.46%, respectively, which are 5–7% higher than those of untreated CF samples. These results are similarly attributed to the photostabilization of TiO_2_ particles on the surface. TiO_2_ absorb ultraviolet light, preventing the ultraviolet light from causingthe degradation of organic molecular bonds in cotton fiber. The volume of TiO_2_ becomes smaller with the continuous absorption of UV light until it disappears. After that, UV light will continue to destroy the chemical bonds of cotton fibers, resulting in the decrease of fiber mechanical properties. These results indicated that in situ preparation of TiO_2_ on the surface of cotton fibers alleviated the fiber photoaging rate.

#### 3.2.4. Thermogravimetric Analysis (TG)

**Figure 12:**
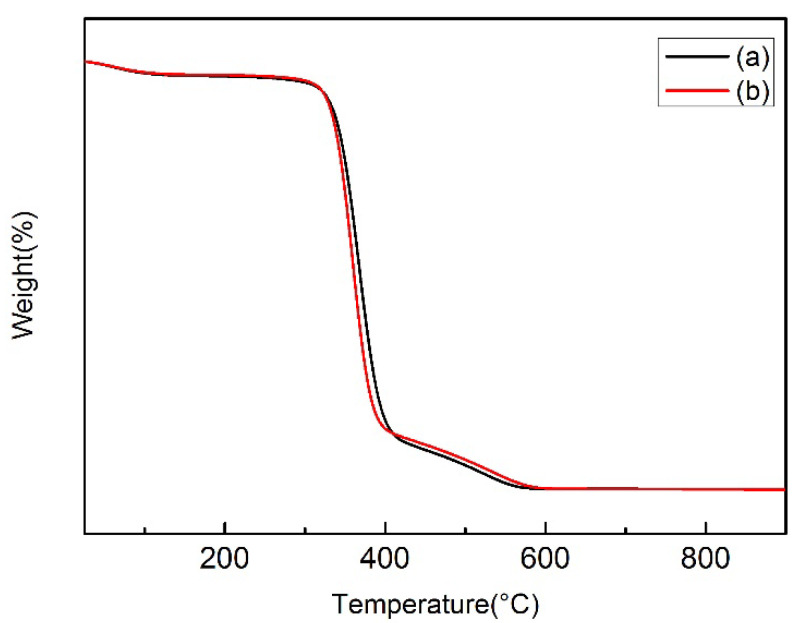
TG curves: (**a**) CF; (**b**) CF-TiO_2_.

Figure 12 shows the thermogravimetric analysis diagram of CF and CF-TiO_2_ samples. The first interval (100–200 °C) is the microweight loss stage, which is mainly the loss process of intermolecular bound water. The second stage is the thermal decomposition stage (400–600 °C), and the start time of fiber decomposition is basically the same. Due to the better thermal stability of TiO_2_, the residual mass of CF-TiO_2_ samples is higher than that of untreated CF fibers. The third zone (600–850 °C) is the stable stage of cotton fiber carbonization. The results show that the synthesis of TiO_2_ on the surface of cotton fibers has little effect on the thermal stability of the fibers.

### 3.3. Illustration of Modification

**Figure 13:**
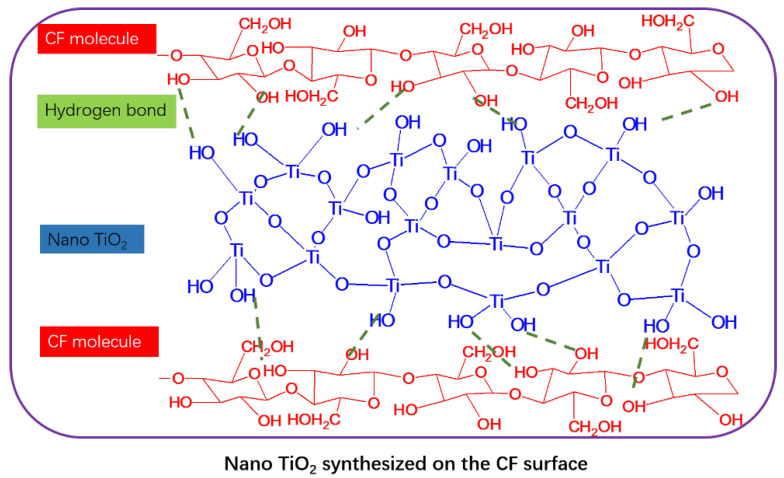
An illustration of the modification in SCCO_2_.

According to the above results and discussion, the model of the modified cotton fiber process is shown in Figure 13. With SCCO_2_ as the carrier, TBT as the TiO_2_ precursor can be dissolved and carried to the surface of cotton fiber and deposited on the surface of the fiber when CO_2_ is decompressed. In the second step, TiO_2_ particles were synthesized on the surface of cotton fiber by ethanol hydrolysis. TiO^2+^ is absorbed by a large number of hydroxyl groups on the surface of cotton fiber, and then formed through hydrolysis and condensation to grow into TiO_2_ particles [35]. In addition, TiO_2_ has hydrogen bonds with the hydroxyl group of CF molecular chains, and provides secondary inter-fiber forces, which contribute to improving mechanical properties.

## 4. Conclusions

In this study, a two-step method for in situ synthesis of TiO_2_ on the surface of cotton fibers was proposed to improve the photoaging resistance of cotton fibers. FTIR, SEM, DEX, and XPS results showed that TiO_2_ particles were successfully prepared on the surface and that the TiO_2_ particles were bonded together by hydrogen bonding by the reaction with hydroxyl groups on the cotton fiber surface. In addition, SEM and mechanical properties test results show that after UV aging simulation of cotton fiber, the surface of CF-TiO_2_ samples is smoother than that of untreated CF samples under the same light time, and the fracture strength and modulus of CF-TiO_2_ samples are higher than that of untreated CF samples. This is because TiO_2_ can absorb UV rays and reduce the absorption of UV rays by the cotton fiber itself. These findings suggest that the process of preparing particles on the surface of cotton fibers assisted by SCCO_2_ is one of the important methods to improve fiber properties and may be a potential application for oxide modified CFs and other natural fibers.

## Figures and Tables

**Table 1:** Chemical atomic compositions on the surface of fibers.

Sample	Atomic Percent (%)			Atomic Ratio	
	C	O	Ti	O/C	Ti/C
CF	84.89	15.11	0	0.1780	0
CF-TiO_2_	77.96	21.09	0.95	0.2705	0.012

**Table 2:** Mechanical properties of CF and CF-TiO_2_ samples.

Samples	Tensile Strength		Tensile Modulus	
	Measured Value /MPa	Standard Deviation	Measured Value /MPa	Standard Deviation
CF	401.06	1.78	2.82	1.83
CF-TiO_2_	426.60	1.33	3.04	1.64

## Data Availability

Not applicable.
